# A *de novo* variant in the *ASPRV1* gene in a dog with ichthyosis

**DOI:** 10.1371/journal.pgen.1006651

**Published:** 2017-03-01

**Authors:** Anina Bauer, Dominik P. Waluk, Arnaud Galichet, Katrin Timm, Vidhya Jagannathan, Beyza S. Sayar, Dominique J. Wiener, Elisabeth Dietschi, Eliane J. Müller, Petra Roosje, Monika M. Welle, Tosso Leeb

**Affiliations:** 1 Institute of Genetics, Vetsuisse Faculty, University of Bern, Bern, Switzerland; 2 DermFocus, University of Bern, Bern, Switzerland; 3 Department of Clinical Research, Molecular Dermatology and Stem Cell Research, University of Bern, Bern, Switzerland; 4 Dermavet, Oberentfelden, Switzerland; 5 Institute of Animal Pathology, Vetsuisse Faculty, University of Bern, Bern, Switzerland; 6 Hubrecht Institute, CT Utrecht, The Netherlands; 7 Clinic for Dermatology, Inselspital, Bern University Hospital, Bern, Switzerland; 8 Division of Clinical Dermatology, Vetsuisse Faculty, University of Bern, Bern, Switzerland; University Medical Center Freiburg, GERMANY

## Abstract

Ichthyoses are a heterogeneous group of inherited cornification disorders characterized by generalized dry skin, scaling and/or hyperkeratosis. Ichthyosis vulgaris is the most common form of ichthyosis in humans and caused by genetic variants in the *FLG* gene encoding filaggrin. Filaggrin is a key player in the formation of the stratum corneum, the uppermost layer of the epidermis and therefore crucial for barrier function. During terminal differentiation of keratinocytes, the precursor profilaggrin is cleaved by several proteases into filaggrin monomers and eventually processed into free amino acids contributing to the hydration of the cornified layer. We studied a German Shepherd dog with a novel form of ichthyosis. Comparing the genome sequence of the affected dog with 288 genomes from genetically diverse non-affected dogs we identified a private heterozygous variant in the *ASPRV1* gene encoding “aspartic peptidase, retroviral-like 1”, which is also known as skin aspartic protease (SASPase). The variant was absent in both parents and therefore due to a *de novo* mutation event. It was a missense variant, c.1052T>C, affecting a conserved residue close to an autoprocessing cleavage site, p.(Leu351Pro). *ASPRV1* encodes a retroviral-like protease involved in profilaggrin-to-filaggrin processing. By immunofluorescence staining we showed that the filaggrin expression pattern was altered in the affected dog. Thus, our findings provide strong evidence that the identified *de novo* variant is causative for the ichthyosis in the affected dog and that ASPRV1 plays an essential role in skin barrier formation. *ASPRV1* is thus a novel candidate gene for unexplained human forms of ichthyoses.

## Author summary

The skin undergoes a constant process of self-renewing and keratinocytes migrate from the basal layer of the epidermis to the uppermost layer, the stratum corneum, as they differentiate. A defect in the differentiation of keratinocytes can lead to cornification disorders such as ichthyosis. The most common form of this disorder in humans is ichthyosis vulgaris caused by variants in the filaggrin gene. Filaggrin is required for bundling intermediate filaments resulting in the flattening of keratinocytes. Filaggrin is produced from profilaggrin and the processing steps involve several enzymes including proteases. In the present study, we sequenced the genome of a dog with a novel form of ichthyosis. By comparing this sequence to 288 control genomes, we identified a private missense variant in the *ASPRV1* gene encoding the retroviral-like aspartic protease 1, also known as SASPase, which is involved in the processing of profilaggrin. The variant was due to a *de novo* mutation event, which is consistent with the patient being an isolated single case of a novel form of ichthyosis. Filaggrin protein expression was altered in the skin of the affected dog. Thus, our results strongly suggest that genetic variants in *ASPRV1* can cause ichthyosis by altering filaggrin processing.

## Introduction

The skin and in particular the epidermis provide both an outward and inward barrier function, which is essential for survival. Aberrant skin development or homeostasis can impair this barrier function and may result in skin disorders. Ichthyoses are a heterogeneous group of skin disorders characterized by dry skin, scaling and/or hyperkeratosis, often associated with erythroderma [1,2].These clinical signs are caused by a defect in the terminal differentiation of keratinocytes and subsequent desquamation taking place in the uppermost layer of the epidermis, the stratum corneum. Ichthyoses are primarily inherited skin disorders that can either be non-syndromic, when clinical findings are limited to the skin, or syndromic in case additional organs are involved [2]. Non-syndromic forms of ichthyoses are further sub-classified into common ichthyoses, autosomal recessive congenital ichthyoses, keratinopathic ichthyoses caused by variants in different keratin genes, and other forms of ichthyoses [1,2].

The common ichthyoses consist of ichthyosis vulgaris (IV) and recessive X-linked ichthyosis (RXLI). IV is the most common and mildest form of ichthyosis with an incidence of approximately 1:250 to 1:1000 in humans [2,3]. IV is caused by different semidominant genetic variants in the *FLG* gene encoding filaggrin [4]. Filaggrin (filament aggregating protein) is a keratin bundling protein and a key player in the formation of the stratum corneum [5]. The precursor of filaggrin is the >400 kDa protein profilaggrin, which is the major component of keratohyalin granules in the granular layer of the skin [6,7]. Profilaggrin consists of a unique N-terminus and a series of filaggrin units separated by short linker peptides. The initially highly phosphorylated profilaggrin is dephosphorylated and cleaved by proteases into individual filaggrin molecules during the cornification process. In addition to its role in aggregating keratin intermediate filaments into bundles, filaggrin is also degraded into free amino acids that contribute to the hydration of the cornified layer [8].

RXLI, sometimes also called X-linked ichthyosis (XLI), is clinically more severe and characterized by dark brown scales and generalized dry skin. It is caused by variants, mainly large deletions, affecting the *STS* gene, which encodes steroid sulfatase [9].

Autosomal recessive congenital ichthyoses (ARCI) are the second category of non-syndromic ichthyoses. They may be caused by variants in at least 9 different genes: *ABCA12*, *ALOX12B*, *ALOXE3*, *CERS3*, *CYP4F22*, *LIPN*, *NIPAL4*, *PNPLA1* and *TGM1* [2].

Finally, keratinopathic ichthyoses, the third category of non-syndromic ichthyoses, are caused by variants in the *KRT1*, *KRT2*, or *KRT10* genes [2]. Thus, there are currently 14 human genes implicated in different forms of non-syndromic ichthyoses [2,10].

Dogs represent valuable models for many human hereditary diseases and enabled for example the discovery of *PNPLA1* as an ichthyosis gene. The first pathogenic *PNPLA1* variant was identified in Golden Retriever ichthyosis, which is characterized by a mild phenotype. Interestingly, this canine genodermatosis currently has an extremely high prevalence in the breed [11,12]. Other dog models for human non-syndromic ichthyoses include Norfolk Terriers with an epidermolytic ichthyosis caused by a *KRT10* variant [13], Bulldogs with ARCI caused by a *NIPAL4* variant [14], and Jack Russell Terriers with another form of ARCI caused by a *TGM1* variant [15]. Further cases of canine ichthyoses have been reported, but the underlying genetic defects have not been solved [16]. Thus, dogs might help to identify additional ichthyosis genes, which might be of relevance for unsolved human forms of ichthyoses.

In the present study, we describe a novel non-epidermolytic form of ichthyosis in a German Shepherd. In this breed, until now, no ichthyosis cases have been reported in the scientific literature. We therefore applied a whole genome sequencing approach to unravel the causative genetic variant.

## Results

### Clinical and histopathological phenotype

**Fig 1:**
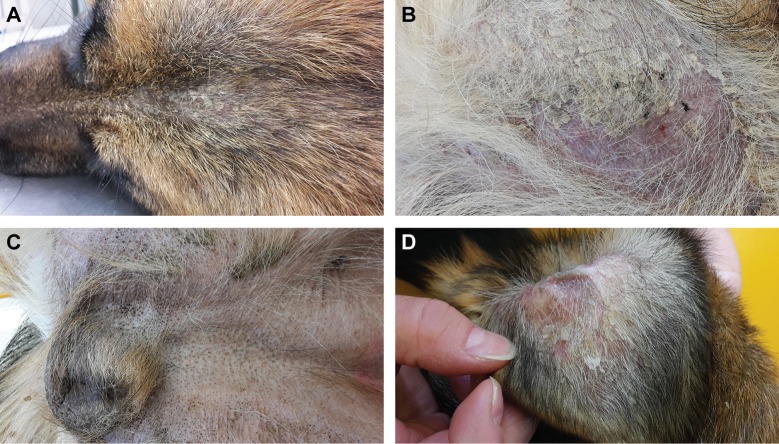
Clinical phenotype of the affected German Shepherd. **(A)** Hypotrichosis and scaly skin. **(B)** Alopecic region of the thigh with erythema and scales. **(C)** Comedones in the inguinal region. **(D)** Pinna with scales and erythema.

An intact female German Shepherd was presented at 10 months of age with a history of severe scaling of the skin with mild pruritus. According to the owner, the lesions started to develop shortly after birth. Dermatological examination revealed generalized hypotrichosis and focal areas of alopecia with generalized severe exfoliation of greyish scales and mild erythema. Comedones were seen on the ventral abdomen and in the perivulvar area (Fig 1).

The owner reported that this phenotype had not been seen in the six littermates or the parents of the affected German Shepherd. The skin condition improved under topical treatment with a rehydrating, anti-seborrheic spray and shampoo.

**Fig 2:**
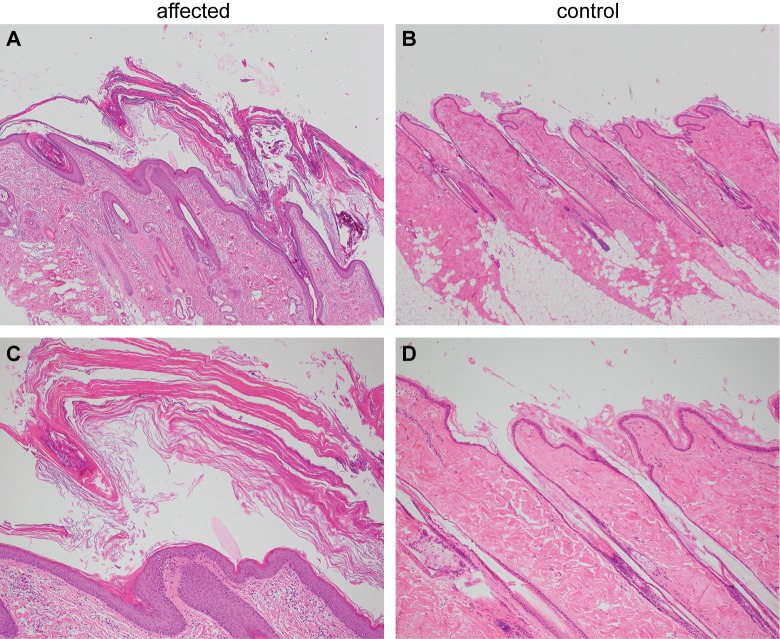
Histopathological findings in skin of the ichthyotic dog and a control dog. **(A)** Skin of the ichthyotic dog with severe laminar to compact orthokeratotic hyperkeratosis extending in the follicular infundibula and covering a mildly hyperplastic epidermis. Hematoxylin and Eosin 40x. **(B)** Skin of a normal dog with normal thickness of the epidermis covered by basket-weave orthokeratotic keratin. Hematoxylin and Eosin 40x. **(C,D)** Skin sections of the same dogs as in A and B at higher magnification (100x).

Histopathological analysis of four skin biopsies from different body regions revealed a severe laminar to compact orthokeratotic hyperkeratosis extending into the follicular infundibula in all biopsies. The keratin layers were multifocally exfoliating as large scales. The underlying epidermis was mildly hyperplastic. In the biopsy from the inguinal region, the infundibula of the hair follicles were moderately dilated. The histological findings were consistent with a cornification disorder and an inherited non-epidermolytic ichthyosis as possible cause (Fig 2).

### Genetic analysis

We sequenced the genome of the affected dog at 31x coverage and called SNVs and small indel variants with respect to the reference genome (CanFam 3.1). We then compared these variants to whole genome sequence data of 288 control dogs of various breeds including 13 German Shepherds not closely related to the affected dog (Table 1). As we did not find any protein-changing variants in the 14 known ichthyosis-associated genes, we hypothesized that the affected dog represented an isolated case of a novel form of ichthyosis. Consequently, we considered both a recessive and a dominant mode of inheritance for the hypothetical mutant allele.

**Table 1:** Variants detected by whole genome re-sequencing of the affected dog.

Filtering step^a^	Number of variants
Homozygous variants in the whole genome	3,108,583
Private homozygous variants (absent from 288 control genomes)	797
Protein-changing private homozygous variants (absent from 288 control genomes)	4
Heterozygous variants in the whole genome	2,767,699
Private heterozygous variants (absent from 288 control genomes)	3,202
Protein-changing private heterozygous variants (absent from 288 control genomes)	19

^a^ The sequences were compared to the reference genome (CanFam 3.1) from a Boxer.

As purebred dogs are maintained in closed populations with a small effective population size and a considerable degree of inbreeding, recessive genetic defects within a breed typically can be traced back to single founders and are mostly found in homozygous state in affected dogs. In a first analysis, assuming a recessive mode of inheritance, we therefore searched for homozygous private protein-changing variants in the affected dog. Our automated pipeline detected 4 such homozygous protein-changing variants. However, upon individual visual inspection all 4 variants turned out to be sequencing artifacts. They were either located close to gaps in the reference assembly, in highly repetitive sequences, or in regions with low read coverage (S1 Table).

Given that the described dog was the only case in a litter of seven and ichthyosis had never before been reported in the German Shepherd breed, we hypothesized that a dominant mode of inheritance due to a *de novo* mutation event was more likely than a recessive mode of inheritance. In a second analysis, we therefore filtered for heterozygous private protein-changing variants. Our automated pipeline identified 19 such variants in 13 genes. None of the identified variants was located in a known ichthyosis gene. In order to identify potential *de novo* variants, we obtained whole genome sequences from both parents of the affected dogs. Inspection of the sequencing data for each of the 19 heterozygous candidate variants revealed that only one of them was indeed a *de novo* variant (S1 Table).

**Fig 3:**
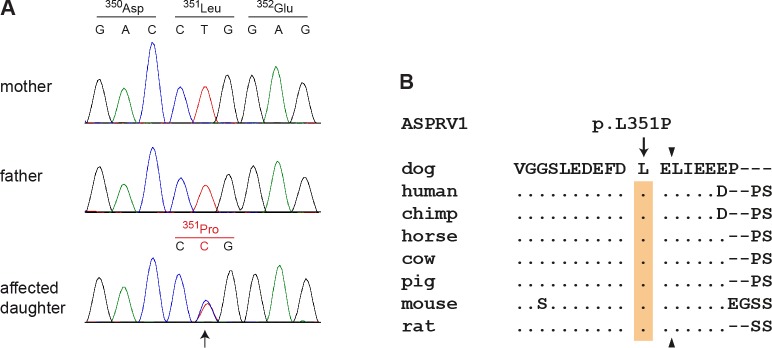
Sanger electropherograms of the ASPRV1:c.1052T>C variant and evolutionary conservation of leucine 351 in the ASPRV1 protein. **(A)** A genomic ASPRV1 fragment was amplified by PCR and sequenced with the Sanger method. The figure shows genotypes of the affected daughter and both her non-affected parents. The position of the variant is indicated by an arrow. Note that the variant C-allele is only present in the daughter, but absent from both parents indicating a *de novo* mutation event. **(B)** The leucine residue at position 351 of the canine ASPRV1 protein is strictly conserved in several species and located close to the C-terminal auto cleavage site, which is indicated by arrowheads. The multiple alignment was done using accessions XP_013972931.1 (*Canis lupus familiaris*), NP_690005.2 (*Homo sapiens*), XP_525777.1 (*Pan troglodytes*), XP_014586589.1 (*Equus caballus*), XP_003586694.1 (*Bos taurus*), XP_003354829.2 (*Sus scrofa*), NP_080690.2 (*Mus musculus*) and XP_008761336.1 (*Rattus norwegicus*). A full-length alignment of the proteins from selected species is given in S1 Fig.

This *de novo* variant was a missense variant, c.1052T>C, located in the *ASPRV1* gene encoding “aspartic peptidase, retroviral-like 1” also known as skin aspartic protease (SASPase), which is involved in profilaggrin-to-filaggrin processing [17,18,22]. We performed Sanger sequencing in the affected dog and both parents and confirmed that the variant was absent in both parents (Fig 3A). We experimentally confirmed the correct parentage by an analysis of microsatellite and SNV genotypes in the trio.

The ASPRV1:c.1052T>C variant is predicted to result in the amino acid substitution p.(Leu351Pro). The leucine at position 351 is strictly conserved among different species of placental mammals and only one residue away from one of the major cleavage sites required for auto-activation of the protein (Fig 3B) [17].

### Functional confirmation

**Fig 4:**
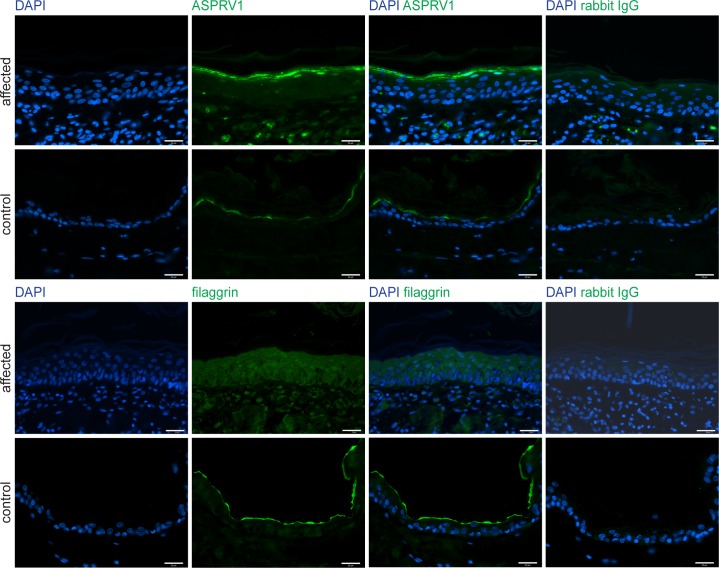
ASPRV1 and filaggrin protein expression. Skin sections of the case and a non-affected control dog were stained for immunofluorescence with an anti-ASPRV1 or anti-filaggrin antibody, respectively. The ASPRV1 signal was stronger in the affected dog than in the control dog. The typical specific expression pattern of filaggrin, a line at the border between stratum granulosum and stratum corneum, was observed in the control dog (bottom panels) but not in the affected dog (third row of panels). Specificity of the antibodies was demonstrated by using rabbit IgG as primary antibody (last column). Scale bars: 25 μm.

To assess the putative impact of the ASPRV1 missense variant we performed immunofluorescence staining with anti-ASPRV1 antibodies on skin sections of the affected and a control dog. The ASPRV1 signal in the affected dog showed the expected localization, mainly in the stratum granulosum, but was stronger than in the control dog (Fig 4). As this experiment could not assess whether the detected ASPRV1 protein is functional, we also investigated filaggrin processing by immunofluorescence staining with anti-filaggrin antibodies. This experiment demonstrated an abnormal filaggrin expression pattern in the affected dog (Fig 4). In the affected dog, diffuse staining across epidermal layers (from stratum basale through stratum spinosum to stratum granulosum) and some nuclear staining indicated defective processing of profilaggrin.

## Discussion

In the present study we identified a *de novo* missense variant in the canine *ASPRV1* gene in a dog with a novel form of ichthyosis. We provide five arguments supporting the causality of the ASPRV1:c1052T>C variant for the observed ichthyosis.

First, the c.1052T>C variant leads to a non-conservative amino acid exchange p.(Leu351Pro) close to the functionally important auto-cleavage site of the ASPRV1 protease. It is thus conceivable that this specific genetic variant might affect the protein function.

Second, the c.1052T>C was absent from 288 non-affected dogs of different breeds and thus perfectly associated with the disease status.

Third, the affected dog was heterozygous for the variant, but the mutant allele was absent in blood leukocytes of both parents. We therefore confirmed that the variant had arisen by a *de novo* mutation event that must have occurred in either one of the parental germlines or during early embryonic development of the affected dog. While exact numbers on the frequencies of *de novo* mutation events in dogs were not available at the time of this study, an analysis of 10 human trios reported 73 *de novo* mutation events on average per trio [19]. In another study on human *de novo* mutation events, it was shown that only ~1.3% of the *de novo* variants actually represented protein-changing variants [20]. Similar numbers of *de novo* mutation events were observed in cattle [21]. If we assume that these numbers are similar in dogs, one might expect roughly one *de novo* protein-changing variant in any dog on average, which exactly matches our data with one identified protein-changing variant in the affected dog. We deem it unlikely that such an event would coincidentally affect a gene with a known role in filaggrin processing without being causative for the ichthyosis phenotype.

Fourth, we observed a difference in the ASPRV1 protein expression between the affected dog and a control dog. Somewhat surprisingly, the ASPRV1 protein expression was upregulated in the affected dog. Such an upregulation might have been caused by a compensatory mechanism, if the expressed ASPRV1 protein is non-functional as has been reported for other missense variants [22]. Thus, the increased ASPRV1 quantity is at least compatible with a causal role of the ASPRV1 missense variant in the observed ichthyosis.

Finally and as fifth argument, we experimentally confirmed that the filaggrin protein expression pattern was altered in the affected dog suggesting a defect in profilaggrin-to-filaggrin processing. In our opinion and taken together, these arguments prove the causality of the ASPRV1:c1052T>C variant for the observed ichthyosis beyond any reasonable doubt.

The *ASPRV1* gene and its encoded protease were initially identified in humans and shortly afterwards in mice. ASPRV1 protein expression was only detected in stratified epithelia and was restricted to the stratum granulosum [17,18]. Further studies suggested that ASPRV1 cleaves the linker sequence in profilaggrin. A deficiency of ASPRV1 resulted in a lower level of stratum corneum hydration [23]. Furthermore, high ASPRV1 protein levels were present in several non-neoplastic skin disorders [17,24].

In transgenic mice, aberrant Asprv1 expression resulted in delayed wound closure [25]. *Asprv1* deficient mice (*Asprv1*^*-/-*^) in a C57BL/6J background showed characteristic parallel skin wrinkles or lined grooves parallel to the body axis, but were reported to have normal skin histology and did not show any signs of ichthyosis [18]. The skin of *Asprv1*^*-/-*^ mice in a hairless background (Hos:HR-1) displayed more fine wrinkles and was drier and rougher than in *Asprv1*^*+/-*^ or *Asprv1*^*+/+*^ mice [23]. In addition to an increased number of epidermal cell layers and a lower level of stratum corneum hydration, a decreased amount of filaggrin monomers together with an accumulation of aberrantly processed profilaggrin in the lower stratum corneum was observed in these mice. The total amount and the composition of free amino acids was however not significantly different from control mice [23].

Our results clearly showed an altered filaggrin expression pattern in the skin of an affected dog with the *ASPRV1* variant. Thus, similar to Asprv1 deficient mice, profilaggrin processing appeared to be defective in the affected dog. In contrast to the findings by Matsui et al. [23], we did however not detect an accumulation of incompletely processed filaggrin in the stratum corneum, but rather in the stratum spinosum and stratum granulosum. It remains unclear why the *Asprv1*^*-/-*^ mice did not show an ichthyosis phenotype as the *ASPRV1* mutant dog. Potential explanations include a gain of function effect of the specific canine missense variant or general differences in the homeostasis of the epidermis between mice and dogs.

According to our knowledge, the *ASPRV1* gene has not been associated with any form of ichthyosis in humans. Missense variants in the human *ASPRV1* gene were reported in 5 of 196 Japanese patients with atopic eczema and 2 of 28 control subjects [23]. Two of the identified variants, p.V243A (identified in a control subject) and p.V187I (identified in 3 atopic eczema patients) led to absence or reduction of ASPRV1 activity *in vitro* [23]. Another study failed to find significant associations between *ASPRV1* genetic variants and atopic eczema or clinically dry skin in different cohorts of Caucasian ancestry [26].

In conclusion, with the identification of a dominant *de novo* missense variant in the *ASPRV1* gene of an ichthyotic dog, we present a new candidate gene for ichthyosis. It seems possible that *ASPRV1* variants might also contribute to unsolved human ichthyoses.

## Materials and methods

### Ethics statement

All animal experiments were performed according to the local regulations. The dogs in this study were examined with the consent of their owners. The study was approved by the “Cantonal Committee For Animal Experiments” (Canton of Bern; permits 22/07, 23/10, and 75/16).

### Clinical and histopathological examination

The affected dog was examined by a board certified veterinary dermatologist in a private specialist clinic and followed up after initiating treatment with a rehydrating, anti-seborrheic spray (Ermidra, Ufamed AG, Sursee, Switzerland) and shampoo (Sebomild P, Virbac AG, Glattbrugg, Switzerland).The absence of a similar phenotype in littermates, parents and ancestors was reported by the owner. Skin biopsies (6 mm) of the case were taken from the flank, thigh, shoulder, and inguinal region and fixed in 10% buffered formalin for 24 hours. Biopsies were processed, embedded in paraffin and sectioned at 4 μm. Skin sections were stained with hematoxylin and eosin. The histopathology was performed by board certified pathologists.

### DNA isolation and parentage confirmation

We isolated genomic DNA from EDTA blood samples of the affected dog and its parents. We confirmed the parentage by two different approaches: A multiplex PCR with 7 fluorescently labeled microsatellites primer pairs was performed for both parents and the case. Allele sizes were determined on an ABI 3730 capillary sequencer (Life Technologies) and analyzed using the GeneMapper software (Life Technologies). Mendelian transmission of the alleles was confirmed at all 7 loci. The three dogs were additionally genotyped for 173,662 SNVs on the illumina canine_HD chip by GeneSeek/Neogen. We evaluated the parentage using the --genome and --mendel commands in plink 1.07 [27]. Both analyses were in agreement with the assumed parentage (<0.001% genome regions with IBD = 0 for mother and father; 26 Mendel errors for the trio).

### Whole genome sequencing of the affected German Shepherd and its parents

Illumina PCR-free TruSeq fragment libraries with insert sizes of 350 bp—400 bp were prepared. For the affected dog, 276 million 2 x 150 bp paired-end reads or 31x coverage were obtained on a HiSeq3000 instrument. The parents were sequenced at 21x coverage. The reads were mapped to the dog reference genome assembly CanFam3.1 and aligned using Burrows-Wheeler Aligner (BWA) version 0.7.5a [28] with default settings. The generated SAM file was converted to a BAM file and the reads were sorted using samtools [29]. Picard tools (http://sourceforge.net/projects/picard/) was used to mark PCR duplicates. To perform local realignments and to produce a cleaned BAM file, we used the Genome Analysis Tool Kit (GATK version 2.4.9, 50) [30]. GATK was also used for base quality recalibration with canine dbsnp data as training set. The sequence data were deposited under the study accession PRJEB16012 at the European Nucleotide Archive. The sample accessions are SAMEA4506895 for the case (DS043), SAMEA72802918 for the sire (DS053) and SAMEA72802168 for the dam (DS051).

### Variant calling

Putative SNVs were identified in each sample individually using GATK HaplotypeCaller in gVCF mode, and subsequently genotyped per-chromosome and genotyped across all samples simultaneously [31]. Filtering was performed using the variant filtration module of GATK. To predict the functional effects of the called variants, SnpEFF [32] software together with the ENSEMBL (version 80) annotation CanFam 3.1 was used. We additionally visually inspected the short read alignments of the functional candidate genes *ABCA12*, *ALOX12B*, *ALOXE3*, *CERS3*, *CYP4F22*, *FLG*, *KRT1*, *KRT10*, *KRT2*, *LIPN*, *NIPAL4*, *PNPLA1*, *STS*, and *TGM1* in the integrative genome viewer [33] to exclude any structural variants in these genes. We also inspected the *CLDN1* gene in the same manner. For variant filtering we used 288 control genomes, which were either publicly available [34] or produced during other projects of our group. A detailed list of these control genomes is given in S2 Table.

### Gene analysis

We used the dog CanFam 3.1 reference genome assembly for all analyses. Numbering within the canine *ASPRV1* gene corresponds to the accessions XM_014117456.1 (mRNA) and XP_013972931.1 (protein). Numbering within the human *ASPRV1* gene corresponds to the accessions NM_152792.2 (mRNA) and NP_690005.2 (protein).

### Sanger sequencing

We used Sanger sequencing to confirm the candidate variant c.1052T>C in *ASPRV1* in the affected dog and its absence in both parents. A 370 bp fragment containing the variant was PCR amplified from genomic DNA using AmpliTaq Gold 360 Master Mix (Life Technologies) and the primers ACCCCAGGGACAGATTAAGG and AGCTGAAGCTGAAGGCAGAG. After treatment with shrimp alkaline phosphatase and endonuclease I, PCR products were directly sequenced on an ABI 3730 capillary sequencer (Life Technologies). We analyzed the Sanger sequence data using the software Sequencher 5.1 (GeneCodes).

### Immunofluorescence staining and fluorescence microscopy

Immunofluorescence staining was performed on formalin-fixed paraffin-embedded skin sections of an age- and sex-matched control dog and the affected dog with some adaptations as described previously [35]. Briefly, tissue sections were deparaffinized using xylene. For ASPRV1 staining, antigens were retrieved in a microwave oven for 20 min at 95°C in sodium citrate buffer (10 mM sodium citrate, pH 6.0). Blocking was performed for 1.5 hours at room temperature (10% goat serum, 1% BSA, 0.1% Triton X-100; 5% cold fish gelatin in PBS). Tissue sections were incubated with a polyclonal rabbit anti-ASPRV1 antibody (1:250, NBP2-33981, Novus Biological) overnight at 4°C and with the secondary goat anti-rabbit Alexa Fluor 488 nm antibody (1:1000, Abcam) for 1 hour at room temperature. DNA was stained with 4’,6-diamidino-2-phenylindole (DAPI) contained in Vectashield Antifade Mounting Medium (Vector Laboratories). For filaggrin staining, antigens were retrieved in a pressure cooker for 15 min in Tris buffer (100 mM) with 5% urea. Blocking was performed for 1.5 hours at room temperature (10% goat serum, 1% BSA, 0.1% Triton X-100; 5% cold fish gelatin in PBS). Tissue sections were incubated with a polyclonal rabbit anti-filaggrin antibody (1:250, PRB-417P-100, Covance) overnight at 4°C and with the secondary goat anti-rabbit Alexa Fluor 488 nm antibody (1:1000, Abcam) for 1 hour at room temperature. DNA was stained with DAPI (1:1000, Sigma Aldrich). Tissue sections serving as negative controls were incubated with rabbit IgG serum. Images were taken with a Nikon Eclipse 80i fluorescence microscope using a Plan Flour x40/10 oil-immersion objective and excitation wavelength of 393 and 488nm. Pictures were captured and further processed using Improvision Open Lab 5.5.2. software.

## Supporting information

S1 Fig ASPRV1 protein alignment. (PDF)

S1 Table Private protein-changing variants in the affected dog. (XLSX)

S2 Table Control dogs used for whole genome sequencing. (XLSX)

## References

[1] Takeichi T, Akiyama M (2016) Inherited ichthyosis: Non-syndromic forms. J Dermatol 43: 242–251. 10.1111/1346-8138.13243 26945532

[2] Oji V, Tadini G, Akiyama M, Blanchet Bardon C, Bodemer C, Bourrat E, et al (2010) Revised nomenclature and classification of inherited ichthyoses: results of the First Ichthyosis Consensus Conference in Soreze 2009. J Am Acad Dermat 63: 607–641. 10.1016/j.jaad.2009.11.020 20643494

[3] Wells RS, Kerr CB (1966) Clinical features of autosomal dominant and sex-linked ichthyosis in an English population. Br Med J 1: 947–950. 20790920 10.1136/bmj.1.5493.947 PMC1844863

[4] Smith FJ, Irvine AD, Terron-Kwiatkowski A, Sandilands A, Campbell LE, Zhao Y, et al (2006) Loss-of-function mutations in the gene encoding filaggrin cause ichthyosis vulgaris. Nat Genet 38: 337–342. 10.1038/ng1743 16444271

[5] Candi E, Schmidt R, Melino G (2005) The cornified envelope: a model of cell death in the skin. Nat Rev Mol Cell Biol 6: 328–340. 10.1038/nrm1619 15803139

[6] Dale BA, Resing KA, Lonsdale-Eccles JD (1985) Filaggrin: a keratin filament associated protein. Ann New York Acad Sci 455: 330–342. 2417519 10.1111/j.1749-6632.1985.tb50420.x

[7] Presland RB, Haydock PV, Fleckman P, Nirunsuksiri W, Dale BA (1992) Characterization of the human epidermal profilaggrin gene. Genomic organization and identification of an S-100-like calcium binding domain at the amino terminus. J Biol Chem 267: 23772–23781. 1429717

[8] Hoste E, Kemperman P, Devos M, Denecker G, Kezic S, Yau N, et al (2011) Caspase-14 is required for filaggrin degradation to natural moisturizing factors in the skin. J Invest Dermatol 131: 2233–2241. 10.1038/jid.2011.153 21654840

[9] Basler E, Grompe M, Parenti G, Yates J, Ballabio A (1992) Identification of point mutations in the steroid sulfatase gene of three patients with X-linked ichthyosis. Am J Hum Genet 50: 483–491. 1539590 PMC1684279

[10] Lemke JR, Kernland-Lang K, Hörtnagel K, Itin P (2014) Monogenic human skin disorders. Dermatol 229:55–64. 10.1159/000362200 25012694

[11] Grall A, Guaguere E, Planchais S, Grond S, Bourrat E, Hausser I, et al (2012) PNPLA1 mutations cause autosomal recessive congenital ichthyosis in golden retriever dogs and humans. Nat Genet 44: 140–147. 10.1038/ng.1056 22246504

[12] Tamamoto-Mochizuki C, Banovic F, Bizikova P, Laprais A, Linder KE, Olivry T (2016) Autosomal recessive congenital ichthyosis due to PNPLA1 mutation in a golden retriever-poodle cross-bred dog and the effect of topical therapy. Vet Dermatol 27: 306–e75. 10.1111/vde.12323 27237723

[13] Credille KM, Barnhart KF, Minor JS, Dunstan RW (2005) Mild recessive epidermolytic hyperkeratosis associated with a novel keratin 10 donor splice-site mutation in a family of Norfolk terrier dogs. Br J Dermatol 153: 51–58. 10.1111/j.1365-2133.2005.06735.x 16029326

[14] Mauldin EA, Wang P, Evans E, Cantner CA, Ferracone JD, Credille KM et al (2015) Autosomal recessive congenital ichthyosis in American Bulldogs is associated with NIPAL4 (ICHTHYIN) deficiency. Vet Pathol 52: 654–662. 10.1177/0300985814551425 25322746 PMC4492690

[15] Credille KM, Minor JS, Barnhart KF, Lee E, Cox ML, Tucker KA, et al (2009) Transglutaminase 1-deficient recessive lamellar ichthyosis associated with a LINE-1 insertion in Jack Russell terrier dogs. Br J Dermatol 161: 265–272. 10.1111/j.1365-2133.2009.09161.x 19438474

[16] Mauldin EA (2013) Canine ichthyosis and related disorders of cornification. Vet Clin North Am Small Anim Pract 43: 89–97. 10.1016/j.cvsm.2012.09.005 23182326 PMC3529142

[17] Bernard D, Mehul B, Thomas-Collignon A, Delattre C, Donovan M, Schmidt R (2005) Identification and characterization of a novel retroviral-like aspartic protease specifically expressed in human epidermis. J Invest Dermatol 125: 278–287. 10.1111/j.0022-202X.2005.23816.x 16098038

[18] Matsui T, Kinoshita-Ida Y, Hayashi-Kisumi F, Hata M, Matsubara K, Chiba M, et al (2006) Mouse homologue of skin-specific retroviral-like aspartic protease involved in wrinkle formation. J Biol Chem 281: 27512–27525. 10.1074/jbc.M603559200 16837463

[19] Besenbacher S, Liu S, Izarzugaza JM, Grove J, Belling K, Bork-Jensen J, et al (2015) Novel variation and *de novo* mutation rates in population-wide *de novo* assembled Danish trios. Nat Comm 6: 5969. 10.1038/ncomms6969 PMC4309431 25597990

[20] Kong A, Frigge ML, Masson G, Besenbacher S, Sulem P, Magnusson G, et al (2012) Rate of *de novo* mutations and the importance of father's age to disease risk. Nature 488: 471–475. 10.1038/nature11396 22914163 PMC3548427

[21] Harland C, Charlier C, Karim L, Cambisano N, Deckers M, Mullaart E, et al. (2016) Frequency of mosaicism points towards mutation-prone early cleavage cell divisions. bioRxiv.

[22] Matsuda A, Kosugi S (1997) A homozygous missense mutation of the sodium/iodide symporter gene causing iodide transport defect. J Clin Endocrinol Metab 82: 3966–3971. 10.1210/jcem.82.12.4425 9398697

[23] Matsui T, Miyamoto K, Kubo A, Kawasaki H, Ebihara T, Hata K, et al (2011) SASPase regulates stratum corneum hydration through profilaggrin-to-filaggrin processing. EMBO Mol Med 3: 320–333. 10.1002/emmm.201100140 21542132 PMC3377080

[24] Rhiemeier V, Breitenbach U, Richter KH, Gebhardt C, Vogt I, Hartenstein B, et al (2006) A novel aspartic proteinase-like gene expressed in stratified epithelia and squamous cell carcinoma of the skin. Am J Pathol 168: 1354–1364. 10.2353/ajpath.2006.050871 16565508 PMC1606566

[25] Hildenbrand M, Rhiemeier V, Hartenstein B, Lahrmann B, Grabe N, Angel P, et al (2010) Impaired skin regeneration and remodeling after cutaneous injury and chemically induced hyperplasia in taps-transgenic mice. J Invest Dermatol 130: 1922–1930. 10.1038/jid.2010.54 20237492

[26] Sandilands A, Brown SJ, Goh CS, Pohler E, Wilson NJ, Campbell LE, et al (2012) Mutations in the SASPase gene (ASPRV1) are not associated with atopic eczema or clinically dry skin. J Invest Dermatol 132: 1507–1510. 10.1038/jid.2011.479 22318384 PMC3378512

[27] Purcell S, Neale B, Todd-Brown K, Thomas L, Ferreira MA, Bender D, et al (2007) PLINK: a tool set for whole-genome association and population-based linkage analyses. Am J Hum Genet 81: 559–575. 10.1086/519795 17701901 PMC1950838

[28] Li H, Durbin R (2009) Fast and accurate short read alignment with Burrows-Wheeler transform. Bioinformatics 25: 1754–1760. 10.1093/bioinformatics/btp324 19451168 PMC2705234

[29] Li H (2011) A statistical framework for SNP calling, mutation discovery, association mapping and population genetical parameter estimation from sequencing data. Bioinformatics 27: 2987–2993. 10.1093/bioinformatics/btr509 21903627 PMC3198575

[30] McKenna A, Hanna M, Banks E, Sivachenko A, Cibulskis K, Kernytsky A, et al (2010) The Genome Analysis Toolkit: a MapReduce framework for analyzing next-generation DNA sequencing data. Genome Res 20: 1297–1303. 10.1101/gr.107524.110 20644199 PMC2928508

[31] Van der Auwera GA, Carneiro MO, Hartl C, Poplin R, Del Angel G, Levy-Moonshine A, et al (2013) From FastQ data to high confidence variant calls: the Genome Analysis Toolkit best practices pipeline. Curr Protoc Bioinformatics 43:11.0.1–33. 25431634 10.1002/0471250953.bi1110s43 PMC4243306

[32] Cingolani P, Platts A, Wang le L, Coon M, Nguyen T, Wang L, et al (2012) A program for annotating and predicting the effects of single nucleotide polymorphisms, SnpEff: SNPs in the genome of Drosophila melanogaster strain w1118; iso-2; iso-3. Fly 6: 80–92. 10.4161/fly.19695 22728672 PMC3679285

[33] Thorvaldsdottir H, Robinson JT, Mesirov JP (2013) Integrative Genomics Viewer (IGV): high-performance genomics data visualization and exploration. Brief Bioinform 14: 178–192. 10.1093/bib/bbs017 22517427 PMC3603213

[34] Bai B, Zhao WM, Tang BX, Wang YQ, Wang L, Zhang Z, et al (2015) DoGSD: the dog and wolf genome SNP database. Nucleic Acids Res 43 (Database issue): D777–783. 10.1093/nar/gku1174 25404132 PMC4383968

[35] Chervet L, Galichet A, McLean WH, Chen H, Suter MM, Roosje PJ, et al (2010) Missing C-terminal filaggrin expression, NFkappaB activation and hyperproliferation identify the dog as a putative model to study epidermal dysfunction in atopic dermatitis. Exp Dermatol. 19: e343–346. 10.1111/j.1600-0625.2010.01109.x 20626465

