# Supplementary material for: A de novo variant in the ASPRV1 gene in a dog with ichthyosis
Source: PLoS Genet. 2017 Mar 1;13(3):e1006651. doi: 10.1371/journal.pgen.1006651 (PMC5352138; doi:10.1371/journal.pgen.1006651)
Supplement: S1 Fig — (PDF) [file pgen.1006651.s001.pdf]

S1 Figure Protein alignment CLUSTAL W (1.83)

```
Canis lupus familiaris   XP_013972931.1   1      MGWSERAPPGPAKCHGHRPGLWPPHTPMSGDQLCLVMSSRCPGSPVVSSWAQKAAVPGPG   60
Rattus norwegicus        XP_008774007.1   1      MR----SPGGPG---------WASKRPSRTSRTQTACLCLCAQQPARHIVSAAFNLSRPG   47
Homo sapiens             NP_690005.2      1      MG----SPGAS----------LGIKKALQSEQATA----LPASAPAVSQPTAPAPSCLPK   42
Mus musculus             NP_080690.2      1      MR----NPGGPG---------WASKRPLQKKQNTA---CLCAQQPARHFVPAPFNSSRQG   44
Bos taurus               XP_003586694.1   1      MG----SRGGPR---------LGIKKALQREQATA----L---RPAVHQTASPGPSSLPT   40
                                                 *                                           *

Canis lupus familiaris   XP_013972931.1  61      LGTCTCPRAPRTCPAPRSVIAPARLCGLLYAACVAAALAQEGSAMAGTGTGTGTGSGTGT   120
Rattus norwegicus        XP_008774007.1  48      KNTAQPT---KPSLS--SVIAPTLFCAFLYLACVTAELPEVSRRMAASGVRSKEG-----   97
Homo sapiens             NP_690005.2     43      AGQVIPTLLREAPFS--SVIAPTLLCGFLFLAWVAAEVPEESSRMAGSGARSEEG-----   95
Mus musculus             NP_080690.2     45      KNTAQPT---EPSLS--SVIAPTLFCAFLYLACVTAELPEVSRRMATSGVRSKEG-----   94
Bos taurus               XP_003586694.1  41      AGTQXNSLLREALFS--SVIALTLLCGFLYLLWVVAAVPEGSRGMAGSGVRSQE------   92
                                                                  ****    *  *    * *        **  *

Canis lupus familiaris   XP_013972931.1 121      EAGPRGLRGERAFVPEPFDGEGVGARAWLHRFEAIGDLNGWDPAARLRLLRGSLRGRALQ   180
Rattus norwegicus        XP_008774007.1  98      -------RREHAFVPEPFNGTNVAPSLWLNRFEVIDDLNHWDHATKLRFLKESLRGDALD   150
Homo sapiens             NP_690005.2     96      -------RRQHAFVPEPFDGANVVPNLWLHSFEVINDLNHWDHITKLRFLKESLRGEALG   148
Mus musculus             NP_080690.2     95      -------RREHAFVPEPFTGTNLAPSLWLHRFEVIDDLNHWDHATKLRFLKESLKGDALD   147
Bos taurus               XP_003586694.1  93      ----------RAFVPEPFDGASVAPHLWLHRFEVINHLNHWDHITKLRFLKESLRGDALG   142
                                                            ******* *       **  ** *  ** **    ** *  ** * **

Canis lupus familiaris   XP_013972931.1 181      TLRALGPEAQADYAAVKAALLQASGGLP------PREIVFANSMGKGYYLKGKIGKVPVR   234
Rattus norwegicus        XP_008774007.1 151      VYNGLDSQAQGDYNTVKEALLKSFGGLGAAHNK-PKEIVFANSMGKGYYLKGKIGHVPVR   209
Homo sapiens             NP_690005.2    149      VYNRLSPQDQGDYGTVKEALLKAFGVPGAAPSHLPKEIVFANSMGKGYYLKGKIGKVPVR   208
Mus musculus             NP_080690.2    148      VYNGLSSQAQGDFSFVKQALLRAFGAPGEAFSE-PEEILFANSMGKGYYLKGKVGHVPVR   206
Bos taurus               XP_003586694.1 143      AFNGLSPEDQGNYEAVKETLLKTFGGPEAAHSHLPKEIVFANSMGKGYYLKGKIGKVPVR   202
                                                     *    *    **  **    *         * ** ************** * ****

Canis lupus familiaris   XP_013972931.1 235      FLVDSGAQVSVVHPSLWEEVTDGDLDTLRPFENVVKVANGAEMKILGIWDTVVSLGKLKL   294
Rattus norwegicus        XP_008774007.1 210      FLVDSGAQVSVVHPALWEEVTDADLDTLRPFDNVVKVANGAEMKILGVWDTEVALGKLKL   269
Homo sapiens             NP_690005.2    209      FLVDSGAQVSVVHPNLWEEVTDGDLDTLQPFENVVKVANGAEMKILGVWDTAVSLGKLKL   268
Mus musculus             NP_080690.2    207      FLVDSGAQVSVVHPALWEEVTDGDLDTLRPFNNVVKVANGAEMKILGVWDTEISLGKTKL   266
Bos taurus               XP_003586694.1 203      FLVDSGAQVSVVHPSLWEEVTDGELDTLRPFENVVKVANGAEMKILGVWDTVVSLGKLKL   262
                                                 ************** *******  **** ** *************** ***   *** **

Canis lupus familiaris   XP_013972931.1 295      KAEFLVANASAEEAIIGTDVLQDHNAVLDFEHRTCTLKGKKFRLLPVGGSLEDEFDLELI   354
Rattus norwegicus        XP_008774007.1 270      EAHFLVANASAEEAIIGTDVLQDHNAVLDFEHRTCTLKGKKFRLLPVGGSLEDEFDLELI   329
Homo sapiens             NP_690005.2    269      KAQFLVANASAEEAIIGTDVLQDHNAILDFEHRTCTLKGKKFRLLPVGGSLEDEFDLELI   328
Mus musculus             NP_080690.2    267      KAEFLVANASAEEAIIGTDVLQDHNAVLDFEHRTCTLKGKKFRLLPVGSSLEDEFDLELI   326
Bos taurus               XP_003586694.1 263      KAAFLVANASAEEAIIGTDVLQDHNAVLDFEHRTCTLKGRKFRLLPVGGSLEDEFDLELI   322
                                                  * *********************** ************ ******** ***********

Canis lupus familiaris   XP_013972931.1 355      EEEP---AEPGAAP-SY   367
Rattus norwegicus        XP_008774007.1 330      EEE--SSAPEA----SH   340
Homo sapiens             NP_690005.2    329      EED--PSSEEGRQELSH   343
Mus musculus             NP_080690.2    327      EEEEGSSAPEG----SH   339
Bos taurus               XP_003586694.1 323      EEE--PSSEEGGQQLSC   337
                                                 **             *
```
